# Diagnostic Accuracy of the Ambulatory EEG vs. Routine EEG for First Single Unprovoked Seizures and Seizure Recurrence: The DX-Seizure Study

**DOI:** 10.3389/fneur.2020.00223

**Published:** 2020-04-09

**Authors:** Lizbeth Hernández-Ronquillo, Lilian Thorpe, Dianne Dash, Tabrez Hussein, Gary Hunter, Karen Waterhouse, Pragma Laboni Roy, Jose F. Téllez-Zenteno

**Affiliations:** ^1^Community Health and Epidemiology, University of Saskatchewan, Saskatoon, SK, Canada; ^2^Division of Neurology, Department of Medicine, University of Saskatchewan, Saskatoon, SK, Canada; ^3^Neurophysiology Laboratory, Royal University Hospital, Saskatoon, SK, Canada; ^4^Neurophysiology Laboratory, BC Children's Hospital, Simon Fraser University, Vancouver, BC, Canada; ^5^Saskatchewan Health Authority, Saskatoon, SK, Canada; ^6^Division of Neurology, Department of Medicine, Lakeridge Health Oshawa, Oshawa, ON, Canada

**Keywords:** ambulatory EEG, seizure recurrence, sensitivity, specificity, predictive values, diagnostic accuracy, epilepsy, single unprovoked seizure

## Abstract

**Background:** The DX-Seizure study aims to evaluate the diagnostic accuracy (sensitivity, specificity, positive predictive value, negative predictive value, and likelihood ratio) of the ambulatory EEG in comparison with the first routine EEG, and a second routine EEG right before the ambulatory EEG, on adult patients with first single unprovoked seizure (FSUS) and define the utility of ambulatory EEG in forecasting seizure recurrence in these patients after 1-year follow-up.

**Methods:** The DX-Seizure study is a prospective cohort of 113 adult patients (≥18-year-old) presenting with FSUS to the Single Seizure Clinic for evaluation. These patients will be assessed by a neurologist/epileptologist with the first routine EEG (referral EEG) and undergo a second routine EEG and ambulatory EEG. The three EEG (first routine EEG as gold standard) will be compared and evaluated their diagnostic accuracy (sensitivity, specificity, positive predictive value, negative predictive value, and likelihood ratios) with respect of epileptiform activity and other abnormalities. One-year follow-up of each patient will be used to assess recurrence of seizures after a FSUS and the utility of the ambulatory EEG to forecast these recurrences.

**Discussion:** To the best of our knowledge, this will be the first study to prospectively examine the use of ambulatory EEG for a FSUS in adults and its use for prediction of recurrence of seizures. The overarching goal is to improve diagnostic accuracy with the use of ambulatory EEG in patients with their FSUS. We anticipate that this will decrease incorrect or uncertain diagnoses with resulting psychological and financial cost to the patient. We also anticipate that an improved method to predicting the recurrence of seizures will reduce the chances of repeated seizures and their consequences.

## Background

Unprovoked epileptic seizures affect between 4 to 6% of the population by age 74 (1, 2). However, only around 30% of the patients who present with a first single unprovoked seizure (FSUS) will have a recurrence of seizures (i.e., epilepsy) in the next 1–2 years (3).

The FSUS is a powerful event that has profound life-altering effects for patients, such as a driver's license restriction, unemployment, injuries, and accidents. Furthermore, the uncertainty about the recurrence of seizures carries a significant social and psychological burden for patients and their families (4).

The evaluation of patients with FSUS is also a very important clinical issue for physicians, who must decide if the FSUS was a truly epileptic seizure or not, whether the patient is a high risk for seizure recurrence, and ultimately whether the patient should be diagnosed with epilepsy, been treated with antiepileptic drugs (AED) (5) and reduce the consequences of recurrent seizure.

To establish the diagnosis of FSUS and/or epilepsy, the physician must start with a rigorous clinical evaluation with an emphasis on common risk factors for seizure recurrence, such as a family history of seizures, personal history of febrile seizure, head trauma, and brain infections among others. Also, recommended by the American Academy of Neurology and American Epilepsy Society guidelines 2015, is the use of tools such as Magnetic Resonance Imaging (MRI) and routine electroencephalogram (EEG) (6).

The routine EEG is a neurophysiological test that has proved valuable in making a prognostic determination of the recurrence of seizures. If abnormalities on the routine EEG are found, such as focal spikes and generalized spike waves, the risk of seizure recurrence increases two times. However, the routine EEG has its limitations as its accuracy to detect abnormalities after a FSUS has a sensitivity of 17% and specificity of 95% (7). Previous studies have shown that some factors affect the accuracy of recording abnormalities in the routine EEG in FSUS, such as early recording (up to 72 h) relative to the index event (8, 9). Among other factors that increase the accuracy of the EEG are sleep deprivation techniques, use of serial EEG studies, and prolonged recording (10).

Ambulatory EEG is a monitoring technique that allows for the recording of continuous EEG for 24–72 h at home, including sleep sample (11). The use of ambulatory EEG has been shown to result in higher yields compared to the routine EEG in detecting abnormalities (11). There is only one study comparing the ambulatory EEG and EEG with sleep deprivation to detect epileptiform discharges on patients with single unprovoked seizures (12). In this study, the authors examined a group of patients who had ambulatory EEG and compared with another group of patients using sleep-deprived EEG. After a year of the FSUS, the diagnosis of epilepsy was established. The authors concluded that the diagnostic accuracy was similar between both modalities. However, this was a retrospective study in two different groups of subjects who may not have been fully comparable. We, therefore, believe that a rigorous study evaluating the ambulatory EEG in patients with FSUSs and its relationship with recurrence is needed. Here we describe the study design and protocol of the DX-Seizure study.

### Purpose of the Study

The overarching goal of this study is to evaluate the diagnostic performance of the ambulatory EEG for a First Single Unprovoked Seizure and seizure recurrence at 1-year follow-up. The DX-Seizure study will have the following aims:

(1) To evaluate the diagnostic accuracy (sensitivity, specificity, Positive Predictive Value, Negative Predictive Value, and Likelihood Ratios) of the ambulatory EEG in comparison with the first routine EEG, and a second routine EEG, on adult patients with first single unprovoked seizure who present to the Single Seizure Clinic at Royal University Hospital in Saskatoon, SK, Canada.
(2) To estimate the risk of further seizures and define the utility of ambulatory EEG in predicting seizure recurrence after 1-year follow-up among adult patients with FSUS who present to the Single Seizure Clinic at Royal University Hospital in Saskatoon, Sk. Canada.

### Hypothesis

The use of ambulatory EEG (24 h) will increase the accuracy to detect the presence of focal spikes, and generalized spike waves compare with first and second routine EEG (30 min) in patients with FSUS due to the longer length of the recording and the inclusion of sleep sample. Moreover, the use of ambulatory EEG in patients with FSUS will allow predicting recurrence of seizures at 1 year of follow-up.

## Methods and Design

### Study Design and Settings

This is a prospective cohort study (divided into two stages) of adult patients presenting with FSUS. In the first stage, we aim to compare and evaluate diagnostic accuracy (sensitivity, specificity, positive predictive value, negative predictive value, and likelihood ratios) of the ambulatory EEG and first and second routine EEG (first EEG will be the gold standard) with respect of focal spikes and generalized spike waves and other abnormalities. In the second stage, we aim to assess the relationship between recurrence of seizures and the presence of focal spikes and generalized spike waves in the ambulatory EEG so as to predict recurrences of seizures after a 1-year follow-up.

#### Study Population

This study includes adult patients (≥18-year-old) referred to the Single Seizure Clinic (SSC), who experienced the first episode of an epileptic seizure.

#### Single Seizure Clinic and Clinical Protocol

The Single Seizure Clinic is localized at the Royal University Hospital in Saskatoon, Saskatchewan, Canada. The SSC was started in 2011 and is the only available clinic in the province that provides urgent assessment and evaluation of possible seizure episodes in an ambulatory setting with the goal of expediting the epileptologist/neurologist assessment. The SSC accepts referral from all physicians and health providers in the province. A specialized nurse triages the referrals. After the triage, dates for the patient's consultation and EEG are scheduled within the next 2 weeks. The mean time between the FSUS and the first assessment by SSC (and first EEG) will be ~23 days (range 2–134 days) (13).

On the morning of the appointment, a routine EEG is obtained. In the afternoon, the patient first meets with the SSC nurse, who, after an interview, fills out a standardized assessment form. The nurse presents the case to an epileptologist/neurologist, and then the epileptologist/neurologist performs a detailed clinical history of the patient, reviews previous exams (CT, blood work, etc.), and performs a neurological examination (13). At this point, the results of the patient's EEG are reviewed by the epileptologist/neurologist. Based on this evaluation, the epileptologist/neurologist may decide if further investigations are required (i.e., neuroimaging, video EEG, Holter monitoring). If further evaluation is deemed necessary, a second appointment is scheduled. Otherwise, the diagnosis is provided to the patient and plans made for either active treatment or further observation. For this study, all patients will be contacted through the SSC, 1 year after the first SSC consult.

#### Patient Information and Consent

During November 2014 to May 2018, patients referred to the SSC due to the clinical suspicion of an epileptic seizure, and for whom the epileptologist/neurologist corroborated the presence of a single unprovoked seizure were consecutively invited to participate in this prospective study. The epileptologist introduced the purpose of the research and implications (i.e., repeat EEG −30 min of recording and 24 h ambulatory EEG) of being a participant in this study. After appropriate written and signed consent was obtained, patients were booked for repeat EEG and ambulatory EEG in the next available date.

#### Inclusion and Exclusion Criteria

All patients with a diagnosis of single unprovoked seizure will be invited to participate in the study and asked for written consent. First single unprovoked seizure is defined according to the International League against Epilepsy as a “first transient occurrence of signs and/or symptoms due to abnormal excessive or synchronous neuronal activity in the brain, in the absent of a identified proximate precipitant factors” (14) or an event of loss of consciousness with signs and symptoms of an epileptic seizure.

Patients will be excluded if (1) they had a suspicious history of previous epileptic seizures, (2) they had a history of alcohol or drug withdrawal seizure, (3) they had a non-epileptic seizures, (4) they had a previous diagnosis of epilepsy, and/or (5) the first seizure was provoked or presented with status epilepticus.

### Measures

#### Clinical and Demographic Information

The demographic information was taken from the routine, standardized intake questionnaire used by the nurse at the SSC. The clinical information, including details of the FSUS, the initial assessment, date of the initial physician referral, date of the initial assessment, impression by the epileptologist, and results of MRI or CT scans were gathered after appropriate patient consent of the patient to participate in the study.

#### Electroencephalograms

Upon study enrollment and after the first SSC‘s routine EEG (30 min), a second routine EEG (30 min) and ambulatory EEG (24 h) will be set up and completed by Canadian board-certified technologist. We anticipate that the time between the first routine EEG and the second routine EEG/ambulatory EEG will be ~2 weeks [37 days between FSUS and second EEG/ambulatory EEG, with a range of 16–148 days (13)]. As the ambulatory EEG is performed right after the second EEG, the electrodes, wrapping, and EEG system are going to be the same for both tests.

The Natus® Brain Monitor Amplifier and Natus® NueroWorks® Software version 7.1, will be used for all routine EEG and Ambulatory studies.

The routine EEG (first and second) and ambulatory EEG will be recorded using standard gold plate 10 mm diameter disk electrodes with a 2 mm center hole, applied using the international 10–20 system of electrode placement, secured with collodion, and wrapped with conform bandage followed with burn netting to decrease artifact and ensure that the electrode placement remained secure. Impedances will be confirmed at 5,000 Ω or less (15).

For the routine EEGs, the photic, and hyperventilation stimulation will be used. Regarding the photic stimulation protocol, the stimulus will be applied during closed and opened eyes. The frequency of photic stimulation will be increased from 1 to 30 Hz. Regarding hyperventilation, the patient will be asked to breathe deeply for 3 min. These procedures are aligned with the minimal standards for EEG in Canada (16). During both routine EEGs, the technologist annotates any events.

Unique to the ambulatory EEG, the events will be recorded by the patient/family in an event log, detailing the specific time, clinical description, and duration of each event. Furthermore, family/patients will be instructed to press the event button attached to the ambulatory system for all their events, including any auras or spells. The “event” button will annotated the events on the EEG record (17).

After the set-up of the ambulatory EEG, the patient will be sent home with the diary and instructions to come back the next day (24 h recording) to end the record and have the electrodes removed.

All data files will be reviewed and interpreted by Canadian board-certified Electroencephalographers/epileptologist. Comparing the three EEG (first routine EEG, second routine EEG, and ambulatory EEG), the first routine EEG will serve as a gold standard to determine sensitivity, specificity, predictive values, and likelihood ratio.

The same reader will read each trio of EEGs (first, second, and ambulatory EEG) according to the following findings of technical quality:

(1) The assessor's overall impression on the EEG record.
(2) Presence of seizures and location.
(3) Presence of focal spike-waves and location.
(4) Presence of generalized spike-wave.
(5) Presence of slowing and location.
(6) Normal.

Clinical information will be available to each reader for all the EEG's.

#### Limitations of the Test

Among the limitations of the test are artifacts. Artifacts are present in every EEG recording (routine and ambulatory). However, artifacts are bound to be more likely as the duration of recording increases, making the ambulatory EEG more susceptible to artifacts as the movements and routine activities introduce excessive artifact limiting the interpretation (17). However, in a previous studies, artifacts did not significantly limited the analysis of the EEG among children neither adults (11, 18, 19). Thus, we will keep track of any “technical unsatisfactory study” and any routine EEG or ambulatory EEG with results as “technically unsatisfactory study,” will be repeated as soon as possible and at the best convenient time for the patient.

#### Treatment With AED

The decision of treatment initiation by the neurologist/epileptologist, in principle, is going to be individualized. Risk factors for seizure recurrence will be considering, such as epileptiform activity in the EEG, CT/MRI lesion (mesial temporal sclerosis, hippocampal atrophy, cortical malformations), history of stroke, severe trauma, and developmental delay. Finally, driving/work restrictions and individual preferences are going to be considered.

The treatment with AED in patients with FSUS reduces the recurrence of seizures in the next 1–2 years (20), affecting the second stage of the study (relationship between abnormal ambulatory EEG and recurrence of seizures). Accordingly, patients with FSUS treated with AED will be considered patients with epilepsy (with recurrence of seizures) for the final analysis.

#### Follow-Up

**Figure 1:**
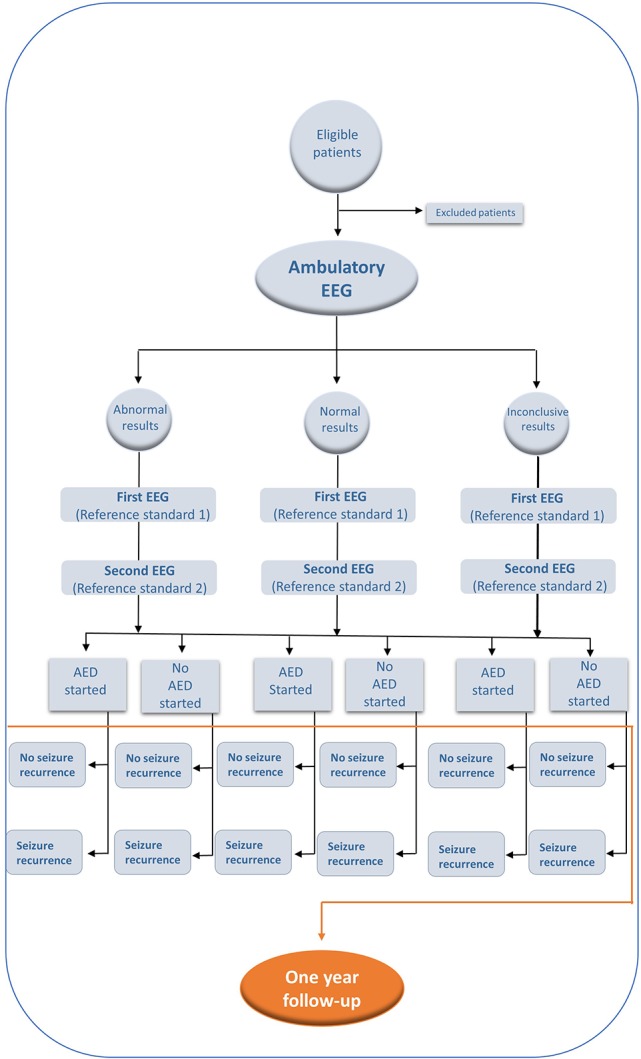
Propose flow diagram for the use of Ambulatory EEG in the diagnosis and recurrance of seizures based in STARD initiative (Standards for Reporting of Diagnostic Accuracy) (21).

After 1 year following the SSC consult, all participants will have a follow-up consult looking for possible seizure recurrence. If a seizure occurs in the first year of follow-up after the patient will be diagnosed as epilepsy (event). Censored cases will be terminated if (1) a person will not experience the event at the end of the study period (1 year); (2) the patient is lost in follow-up during the study period, or (3) the patient withdraws from the study because of death or request by the patient to terminate participation in the study (see Figure 1).

#### Ethical Approval

Ethical approval was obtained from the Biomedical Research Ethics Board at the University of Saskatchewan (#14-30).

## Analysis

### Sample Size

The sample size was calculated for cohort studies with an expected recurrence of seizures of 30%, with assumed hazard ratio of 2.0 (1), confidence level of 95%, and power of 80%. The total sample size was initially planned to be 80 patients. However, this number was increased by 15% for a Cox Proportional model with a hazard ratio between 0.5 and 2.0 (1). As a result, a sample size of 94 patients was assumed to be adequate at this point. Considering possible follow-up losses in this prospective study, we added 20% more patients to a final total recruitment number of 113 patients.

#### Outcome Events

There is one primary outcome variable that is the “recurrence of seizures.” However, the phenomenon of “recurrence of seizures” may have two presentations: (a). the subsequent recurrence of seizures during the 1-year follow-up and (b) clinical diagnosis of epilepsy and initiation of treatment with antiepileptic drugs (AED).

For the second outcome that is the recurrence of seizures during the 1-year follow-up, we will use Cox Proportional Hazard modeling to assess the subsequent seizure, adjusting for variables related to recurrence of seizures, including results of fist routine EEG, second routine EEG, and ambulatory EEG.

#### Independent Variables for Recurrence of Seizures

Age: Previous studies have shown that older patients have a recurrence of seizures between 45 and 62% at 1 year after diagnosis (22).
Sex: Male patients have a higher recurrence of seizures than female patients (1).
Presence of nocturnal seizure: the presence of nocturnal seizures increases the risk of recurrence of seizures (6).
EEG: the presence of spike-wave abnormalities in the EEG (6).
Abnormal CT: cerebral atrophy, arachnoid cyst, and evidence of subclinical cerebrovascular diseases (6, 23).
Abnormal MRI: leukomalacia/gliosis, encephalomalacia, any gray matter lesion, mass lesion, hemorrhage, vascular lesion, hippocampal abnormality, ventricular enlargement >1.5 cm, or prominence of extra-axial fluid spaces >1.0 cm (24).

#### Data Collection and Data Entry

We established a permanent and secure storage system for all the original files, including results of EEGs, SSC notes (copies), and collection tools for each patient accordantly with the ethics requirements.

The data will be entered into the Statistical Package for Social Sciences IBM@ SPSS statistics@ version 24. We will use a double-data entry, followed up with a comparison to check for inconsistencies.

#### Data Analysis

The analysis of the data will be as follow:

#### Diagnostic Accuracy

The diagnostic accuracy will be measure as sensitivity, specificity, positive predictive value (PPV), negative predictive value (NPPV), and likelihood ratio (LR), taking the first routine EEG as the reference standard with 2 × 2 tables and using ROC curves for each EEG modality for comparison. The second EEG will be analyzed with parallel interpretation, individuals that test positive to first EEG, second EEG, or both tests will be considered test positive. This result will be a second accuracy analysis (25).

#### Risk Factors for Seizure Recurrence

The time until a recurrence of seizure will be determined at 1-year follow-up by survival analysis following the next steps (26):

##### Descriptive statistics

Percentage, totals, medians with dispersion analysis will be used to characterize the population under study.

Univariate Kaplan Meier statistics will be used to calculate unadjusted survival, and the log-rank test will be used to compared survival curves. An univariate semi-parametric survival model will be used to identify significance of each predictor.

Multivariate model (Cox Proportional Hazard model) will initially include all covariates (including factor that were non-significant in the univariate analysis, but essential for causality explanation), with sequential removal of non-significant (*p* > 0.05) covariates using likelihood ratio statistics. There will be assessment of potential confounding and interactions.

We will investigate the assumption of the fitted model by two methods: incorporating the interaction between covariates and time in the model and examining covariate-wise residuals (Martingale residuals and Schoenfeld residuals).

We will use SPSS version 24 for diagnostic accuracy and SAS university edition for survival analysis.

#### Management of Missing Data

We will keep careful track of the reasons for missing results (indeterminate test) for every neurophysiology report. In addition, we will use analytic methods to distinguish the type of missing data. Finally, multiple imputation methods will be used for all missing data (27).

## Discussion

To the best of our knowledge, this will be the first study to prospectively examine the use of ambulatory EEG for FSUS in adults and its use for prediction of recurrence of seizures. The overarching goal is to improve diagnostic accuracy with the use of ambulatory EEG in patients with their FSUS. We anticipate that this will decrease incorrect or uncertain diagnoses with resulting psychological and financial cost to the patient. We also anticipate that an improved method to predicting the recurrence of seizures will reduce the chances of repeated seizures and their consequences.

## Data Availability Statement

The data that support this study are available from the corresponding author (LH-R), upon reasonable request.

## Ethics Statement

The studies involving human participants were reviewed and approved by University of Saskatchewan Ethics Review Board #14-30. The patients/participants provided their written informed consent to participate in this study. Written informed consent was obtained from the individual(s) for the publication of any potentially identifiable images or data included in this article.

## Author Contributions

LH-R, LT, DD, and JT-Z contributed to the design of the study protocol. DD, TH, KW, and GH carry out activities related with neurophysiology and SSC. PL assisted with data collection. LH-R drafted the manuscript. All authors read and approved the final manuscript.

### Conflict of Interest

The authors declare that the research was conducted in the absence of any commercial or financial relationships that could be construed as a potential conflict of interest.

## Abbreviations

AED: antiepileptic drug
EEG: electroencephalogram
MRI: magnetic resonance imaging
NPV: negative predictive value
PPV: positive predictive value
SSC: single seizure clinic
FSUS: first single unprovoked seizure.
